# High-resolution electron–multi-ion coincidence set-up for gas-phase experiments in the tender and hard X-ray range

**DOI:** 10.1107/S1600577525004862

**Published:** 2025-06-23

**Authors:** Edwin Kukk, Regis Vacheresse, Iyas Ismail, Tatiana Marchenko, Renaud Guillemin, Maria Novella Piancastelli, Marc Simon, Oksana Travnikova

**Affiliations:** a Department of Physics and Astronomy University of Turku FI-20014Turku Finland; b Sorbonne Université CNRS, Laboratoire de Chimie Physique-Matière et Rayonnement F-75005Paris France; ESRF – The European Synchrotron, France

**Keywords:** multi-particle coincidence spectroscopy, deep-core ionization, X-ray induced fragmentation, Auger cascades, gas-phase ionization processes

## Abstract

The MUSTACHE setup (MUlti-STep photofragmentation studies by Auger electron–ion Coincidences using High Energy photons) is a high-resolution electron–multi-ion coincidence system optimized for gas-phase experiments in the tender (∼2–10 keV) and hard (>5 keV) X-ray range. The system integrates a high-resolution hemispherical electron analyzer with a Wiley–McLaren-type ion time-of-flight (TOF) spectrometer, enabling coincidence measurements of Auger electrons and high-energy photoelectrons. Designed to overcome challenges in high-energy electron detection while maintaining excellent energy resolution, the setup covers a broad kinetic energy range up to 5 keV, allowing investigation of hard-X-ray-induced Auger cascades in molecules containing high-*Z* elements, where initial fluorescence decay is followed by Auger processes within this 5 keV detection window. The ion TOF spectrometer provides high-resolution ion mass and momentum analysis, essential for studying light and fast ions generated by deep-core ionization. System capabilities are demonstrated through test measurements on benchmark atomic and molecular systems, such as argon, nitrogen and carbon disulfide. These measurements demonstrate energy-resolved high-kinetic-energy photoelectron–ion coincidences and momentum-resolved multi-ion coincidences following deep-core ionization and Coulomb explosion. MUSTACHE enables investigations into deep-core ionization, Auger cascade processes and Coulomb explosion dynamics in isolated gas-phase species, offering insights into fundamental ionization and fragmentation processes. These results demonstrate that the MUSTACHE setup is a powerful tool for high-resolution electron–ion coincidence spectroscopy, extending advanced coincidence techniques into the hard X-ray regime and providing unprecedented opportunities for studying high-energy X-ray-induced phenomena.

## Abstract 2

The MUSTACHE setup, described in this work, provides a state-of-the-art approach for investigating phenomena induced by high-energy X-rays, specifically targeting deep-core ionization, Auger cascades, and molecular photofragmentation triggered by these processes. Optimized for the tender and hard X-ray range, this high-resolution electron–multi-ion coincidence setup enables efficient detection of high-kinetic-energy electrons and their correlated ions with high momentum resolution.

## 1. Introduction

The rapid evolution of X-ray and ultraviolet light sources in recent decades, such as the latest generation of synchrotrons and free-electron lasers, as well as high-harmonic-generation sources, has motivated an active development of spectroscopic methods and equipment. Coincidence methods, that record several particles emitted as a result of a photoinduced event, have become a mainstream spectroscopic tool at such X-ray light sources. Coincidence measurements are especially useful in small quantum systems such as single molecules in the gas phase, where they provide detailed and diverse information about the events following photoabsorption. In this work, we will describe one such coincidence method and experimental set-up, namely that for electron–multi-ion coincidence measurements. It is commonly referred to as the photoelectron–photoion coincidence (PEPICO) technique; here we emphasize the multi-ion capability by using the acronym PEPI(PI)CO.

Various types of instrumentation can perform PEPI(PI)CO measurements, each particularly suited for collecting specific information such as ion momenta, electron kinetic energy and/or angular distribution, ion masses with high resolution, *etc*. Modern PEPI(PI)CO techniques started to be developed by Danby, Eland and others (Danby & Eland, 1972 ▸; Eland *et al.*, 1986 ▸; Simon *et al.*, 1991 ▸) and are discussed in more recent reviews (*e.g.* Continetti, 2001 ▸; Arion & Hergenhahn, 2015 ▸; Zettergren *et al.*, 2021 ▸). Coincidence setups can be distinguished by the method of electron detection – by an electron time-of-flight (TOF) spectrometer (Hemmers *et al.*, 1998 ▸; De Fanis *et al.*, 2022 ▸), magnetic bottle (Tsuboi *et al.*, 1988 ▸; Mucke *et al.*, 2012 ▸; Strobel *et al.*, 2019 ▸; Penent *et al.*, 2005 ▸), hemispherical analyzer (Ferrand-Tanaka *et al.*, 1996 ▸; Prümper *et al.*, 2007*a* ▸; Kukk *et al.*, 2007 ▸; Kooser *et al.*, 2020 ▸), toroidal analyzer (Céolin *et al.*, 2004 ▸), COLd Target Recoil Ion Momentum Spectrometer (COLTRIMS) (Ullrich *et al.*, 2003 ▸; Jahnke *et al.*, 2004 ▸; Kastirke *et al.*, 2015 ▸; Schmidt–Böcking *et al.*, 2021 ▸) *etc*. The latter has become very popular in studying small quantum systems at free-electron lasers, owing to its superior electron collection efficiency and good ion momentum resolution (Kastirke *et al.*, 2015 ▸). On the other hand, the advantage of the hemispherical electron analyzer lies in its high and easily adjustable electron energy resolution over a broad kinetic energy range, with superior performance at high energies.

In practice, PEPI(PI)CO setups are optimized for a particular set of conditions, such as the expected ion mass, ion kinetic energy and electron kinetic energy ranges, and the number of ionic fragments per event. The intended light source and the photon energy range are common denominators in setting these conditions. Here, we will focus on the *tender*X-ray energies, from about 2 to 10 keV (Piancastelli *et al.*, 2020 ▸).

Retaining high electron energy resolution in coincidence detection is often very desirable since it allows for the accurate determination of energy states of the system, at the beginning or at intermediate steps of the photoinduced dynamics. Hemispherical electron analyzers are arguably the most versatile and common high-resolution electron spectrometers, owing to the extensive adjustability of their settings. They can also be incorporated into PEPI(PI)CO setups, although in this role they have a drawback – their relatively small solid angle of collection can make obtaining high-quality coincidence datasets in an acceptable acquisition time a challenge. Due to the decreasing transmission of the hemispherical analyzer for high-kinetic-energy electrons, implementation of the high-resolution PEPI(PI)CO detection scheme has typically been restricted for UV- and soft X-ray ionization; we are not aware of setups designed for the tender X-ray range. Below, we present a technical description of such a setup, MUSTACHE (MUlti-STep photofragmentation studies by Auger electron–ion Coincidences using High Energy photons). The technical description is followed by test results on various relatively simple and well known systems, chosen to illustrate the performance of MUSTACHE under the intended typical conditions.

## 2. Main components, design and ray-tracing simulations

**Figure 1:**
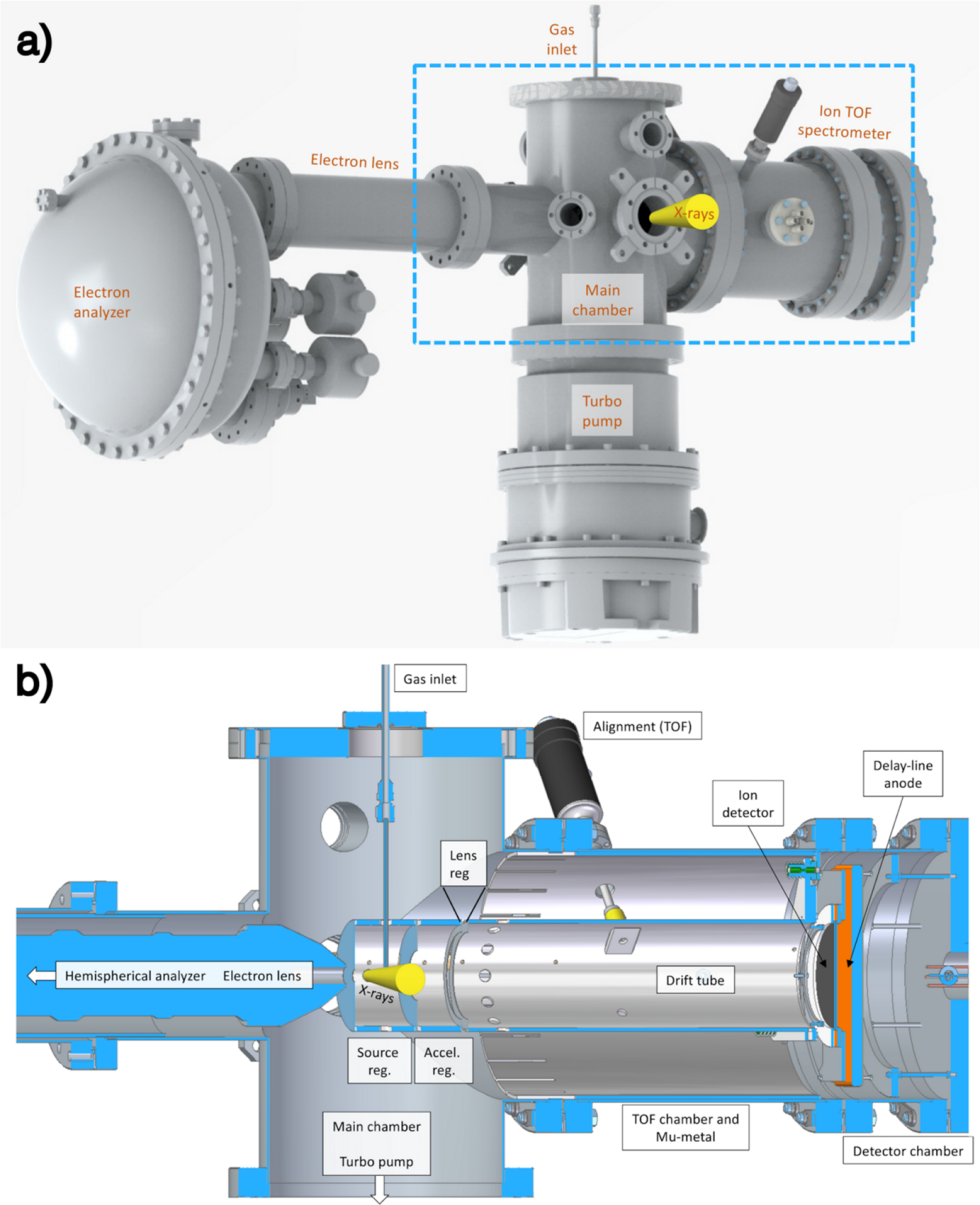
(*a*) Rendering of the main components of the MUSTACHE setup and (*b*) cut-out view of the main chamber and the ion TOF spectrometer, in the region marked by the dashed rectangle in (*a*).

The principal components of the MUSTACHE setup are shown in Fig. 1 ▸(*a*). The electron analyzer [Scienta Omicron DA20(R) HE DLD] and the homemade Wiley–McLaren-type ion TOF spectrometer are mounted on the same axis, defined by the electron lens and the ion-optical elements that face each other across the interaction region in the main chamber. We will refer to this axis as the *main* axis of the system. The electron spectrometer is a hemispherical analyzer with a 135 mm mean radius, equipped with an electron lens capable of collecting electrons up to 5 keV kinetic energy. The lens and analyzer elements can operate in two pre-set voltage settings, the ‘XPS’ and the ‘HAXPES’ mode. The former covers kinetic energies from 2 to 4000 eV and the latter from 1345 to 5000 eV. Pass energies from 20 to 200 eV are available for the XPS and from 200 to 500 eV for the HAXPES mode, although each value does not cover the entire kinetic energy range. Combining different pass energies with the entrance slit width from 0.2 to 1.5 mm, we obtained instrumental linewidths from 13 meV to 1.2 eV (full width at half-maximum). The spectrometer is equipped with a 40 mm multichannel plate (MCP) stack and a two-layer delay-line anode (Elettra DLD) for position-sensitive electron detection. The delay-line detector is suitable for coincidence measurements due to its fast response time (Cautero *et al.*, 2008 ▸).

**Figure 2:**
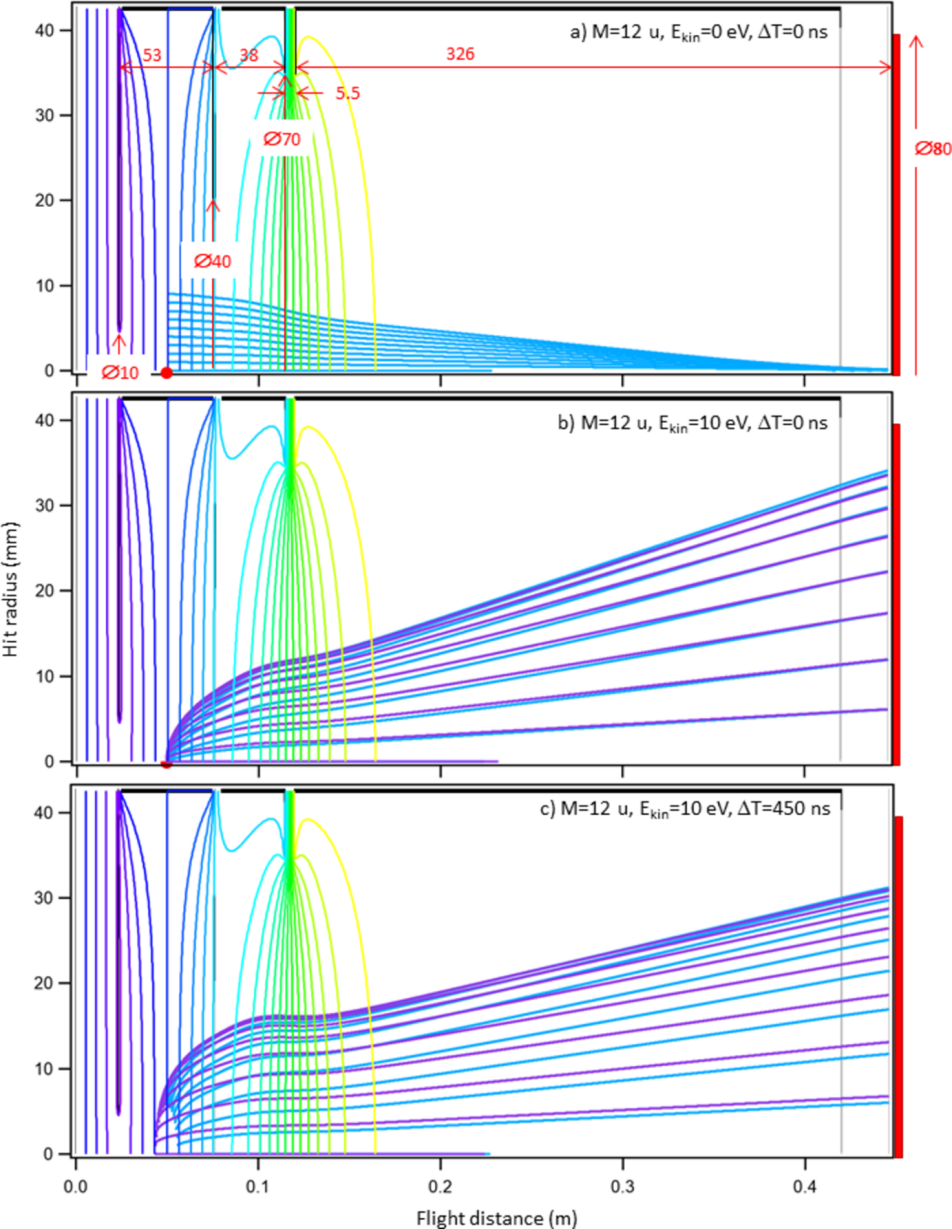
Ray-tracing simulations of C+ atomic ion flight paths, using the extraction pulse of ±420 V, acceleration voltage of −1750 V and ‘lens’ voltage of −400 V. Cross sections of the cylindrical symmetry are shown, with the dimensions (in mm) of the main regions and apertures marked in red. Panel (*a*) shows the spatial (radial) imaging of the source region. Panel (*b*) shows ions with initial kinetic energy of 10 eV, emitted at various angles without extraction pulse delay and (*c*) shows the 10 eV ions with a 450 ns extraction pulse delay. The blue and violet colors of the ion path mark ions initially flying towards and away from the detector, respectively. The colored vertical-curved lines mark equipotential surfaces. Note that the *X*- and *Y*-axes are not on equal scale.

The ion TOF spectrometer is a modified Wiley–McLaren design (Wiley & McLaren, 1955 ▸), optimized particularly for the detection of fast and relatively light (*e.g.* atomic) ions that are common products of deep-core ionization of small molecules. For this purpose, the total flight path was kept relatively short (396 mm) and a large-diameter (80 mm) MCP detector was used. Furthermore, the spectrometer contains an additional optical element, the ‘lens’ [see Fig. 1 ▸(*b*)]. Such an element has been used in earlier designs in order to improve the fast ion collection efficiency and for improved velocity imaging (Lebech *et al.*, 2002 ▸; Prümper *et al.*, 2007*b* ▸; Bomme *et al.*, 2013 ▸; Khan *et al.*, 2015 ▸). The lens has a minor effect on ion flight time and does not interfere with the space focusing (minimization of the dependency of ion TOF from the dimensions of the source volume) properties, but can be used to reduce the lateral spread of fast ions while retaining sufficiently long flight times. Additionally, the lens element can be used to change the spatial magnification of the radial dimension of the source, while keeping the Wiley–McLaren space-focusing condition satisfied. The latter determines the ratio of the extraction and acceleration voltages, and reduces the effect of the longitudinal source size along the spectrometer’s axis. The dimensions of various regions in the TOF spectrometer are given in Fig. 2 ▸(*a*). The ion spectrometer is primarily operated in a pulsed ion extraction field mode, where the pulse is generated either by a constant-frequency pulse generator in a stand-alone mass spectroscopy mode or by the electron detector, when in coincidence mode.

The source region, shared by the electron and ion spectrometers, is an open design, *i.e.* not a higher-pressure region such as a gas cell. Instead, the sample gas is introduced close to the intersection of the X-ray beam and the main axis, in the basic setup through a 500 µm-diameter needle mounted on an *XYZ* manipulator. The source region is a cylinder fixed on the acceleration region, which in turn is fixed on the drift tube [see Fig. 1 ▸(*b*)]. The source region, coaxial with the main axis, contains lateral 10 mm apertures for the sample jet and photon beam. The alignment process begins relative to the electron lens that defines the main axis and the ion TOF assembly is aligned to it using two linear alignment manipulators [one is visible in Fig. 1 ▸(*b*)]. Next, the photon beam is aligned to traverse the source region and intersect the main axis; this is achieved by *XYZ* alignment of the stand of the main chamber using stepper motors. Lastly, the sample jet is aligned to the intersection point of the photon beam and main axis by its own *XYZ* manipulator (not shown in Fig. 1 ▸). The entire setup can also be rotated around the photon beam axis, though it is typically operated with the spectrometers in the horizontal position.

The sample jet exits the source area after crossing the beam and is directed towards the 1000 l s^−1^ turbo pump. The source region has a 10 mm-diameter open aperture towards the electron spectrometer [see also Fig. 2 ▸(*a*)] and a grid-covered 40 mm-diameter aperture towards the acceleration region of the ion TOF spectrometer. The grid is necessary to reduce the penetration of the constantly present acceleration field into the source region when the extraction pulse is not present and the setup is waiting for the next trigger. In the electron–ion coincidence mode, the electrons must have a field-free path through the aperture and into the electron lens during this period, since potential gradients in the source region are directly and negatively reflected in electron energy resolution. This is also the reason why the pulsed extraction field is used in the coincidence mode. The lens element is also beneficial in reducing the field penetration. The pulsed field presents several drawbacks, such as:

(i) The rising edge of the high-voltage (HV) pulse creates a very strong electronic noise in the sensitive electronics of the ion detector. The dampening time of the noise (from reflected pulses) is reduced by mounting the HV pulse generator as close as possible to the electrodes so that the spectrometer can detect the lightest atomic fragment, H^+^. However, when very energetic heavier ions also need to be detected, shorter flight times can be necessary in order to reduce their lateral spread. Shorter flight times necessitate larger extraction and acceleration voltages that in turn increase the noise duration, at the same time reducing the flight time for the H^+^ ions, eventually rendering their detection impossible. Using the lens element allows for more flexibility and, during test measurements, the system was able to reach 4π transmission for C^+^ ions with about 15 eV kinetic energy while still detecting H^+^ fragments.

(ii) When the extraction pulse is triggered by the electron detector in the coincidence mode, an inevitable delay arises between the ionization event and the full voltage of the acceleration pulse. This delay has three main components: electron’s flight time in the main chamber and electron lens, electron’s flight time in the analyzer, and the electronic delay for generating the HV pulse. The former two depend on the voltage settings of the analyzer but are typically about 150 ns for the first two combined and about 300 ns for the third. The approximately 450 ns delay has minimal effect in the cases of low-kinetic-energy-release processes of cationic dissociation, for example, but can be severe in the cases of high-charge Coulomb explosions. The source region was designed with larger dimensions than necessary for a typical Wiley–McLaren design, in order to minimize the detrimental effects of the source volume’s ‘expansion’ during the pulse delay.

Ion ray-tracing simulations aid in choosing the operating parameters, estimating and possibly correcting for effects such as ion loss. Fig. 2 ▸ presents such a simulation for atomic carbon cations. First, panel (*a*) illustrates imaging of finite-sized source region with zero-energy ions, and shows how the chosen lens voltage minimizes the source size’s effect on radial momentum imaging. The extraction and acceleration voltages, on the other hand, were chosen to satisfy the Wiley–McLaren condition (Wiley & McLaren, 1955 ▸), thereby minimizing the source size’s effect on ion flight times. In practice, however, fine-tuning these values by direct measurement can be beneficial, since simulations do not account for effects such as field penetration through the extraction grid or the rise time and fluctuations of the extraction pulse. Panel (*b*) shows an ideal case of collecting 10 eV kinetic-energy ions, with no extraction pulse delay after the ionization. It shows suitable settings for momentum imaging, *e.g.* focusing the ions with ±φ departure angles at the same radius. Panel (*c*) shows the detrimental effects of the extraction pulse delay, with the pre-extraction ‘expansion’ of the source visible at the beginning of the ion trajectories.

The eventual ion detection quantum efficiency *q* for ions that reached the MCP surface is determined by a number of factors. Firstly, the two grids, one in the extractor aperture and the other in front of the MCP (in order to keep electric field penetration into the drift tube) have 90% transmission, and the funnel-type MCP plates from Hamamatsu Corporation have 90% open-area ratio. The theoretical maximum is therefore *q* = 0.73. However, noise discrimination of the MCP voltage pulse additionally removes a fraction of pulses and, if the ion position information is also required, further ion pulse loss occurs in the six anode signals. These losses depend on the condition of the MCP, operating voltages, noise levels in the environment *etc.* Ions can also be rejected by the processing algorithm combining the seven signals. Therefore, we will not quantify the ion detection efficiency for MUSTACHE except for giving the upper limit, but in practice *q* is typically no higher than 0.5.

## 3. Electronics and data acquisition

**Figure 3:**
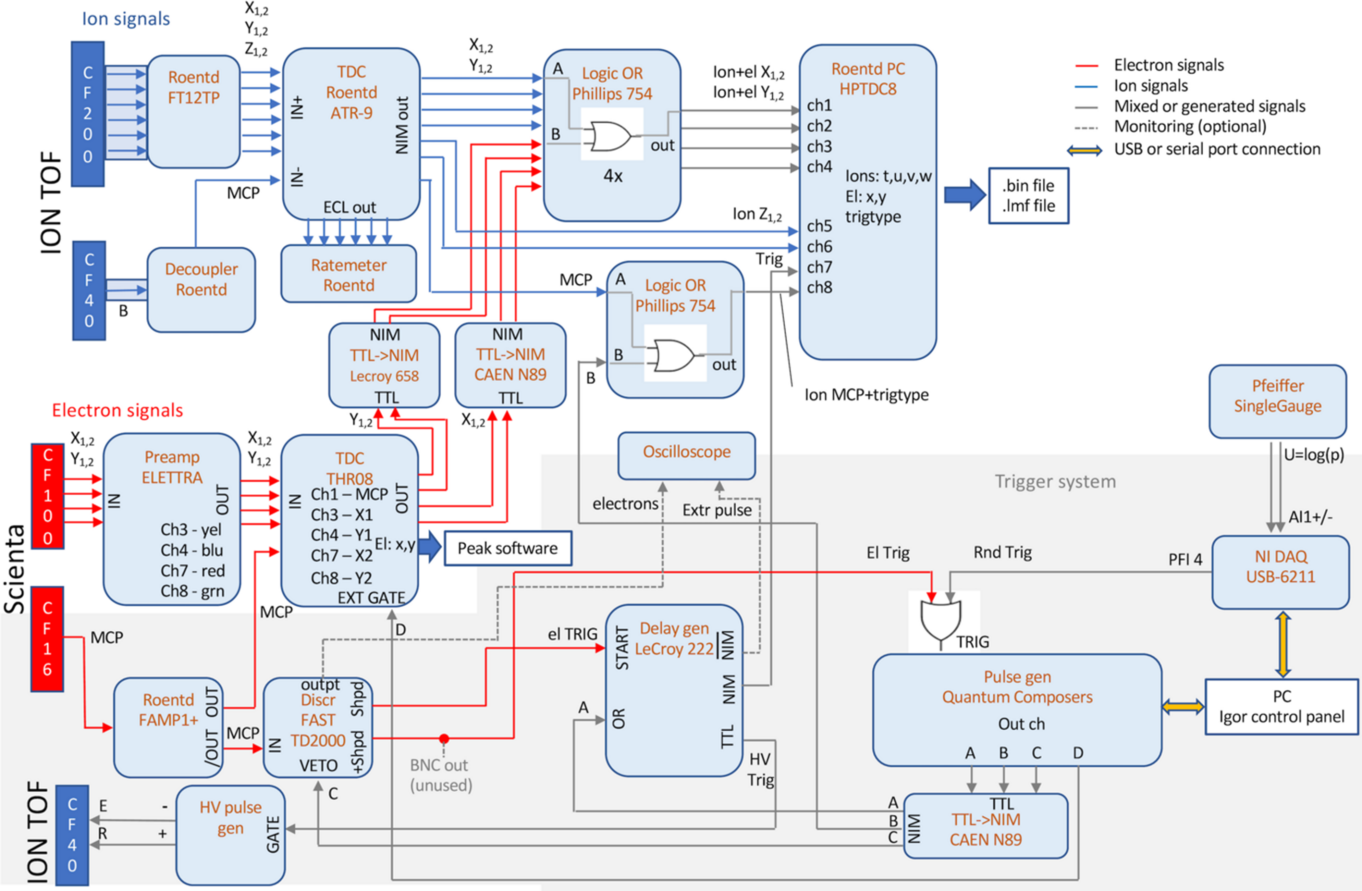
Schematic diagram of the electronic components, signal routes and data acquisition of MUSTACHE.

The electronics and acquisition schematic diagram for electron, ion and coincidence measurements is shown in Fig. 3 ▸. It is divided into the electron (red), ion (blue) and trigger (gray + gray background) subsystems. Let us first consider the independent operation of electron and ion spectrometers.

Electrons detected by the delay-line detector of the Scienta Omicron hemispherical analyzer provide four hit position timing signals X_1,2_, Y_1,2_ from the delay lines and the electron arrival signal from the multichannel plate. After preamplification, these signals are fed into the THR08 time-to-digital converter unit, with its digital output sent to the instrument control PC running the proprietary *PEAK* software. The latter handles the construction of the electron energy spectrum and controls (and scans) the instrument voltages.

Ions, detected by the Roendtek 80 mm HEX (hexagonal) delay-line detector of the TOF spectrometer, provide six position signals *X*_1,2_, *Y*_1,2_, *W*_1,2_ and a single MCP signal. These are preamplified and fed to the ATR-9 time-to-digital converter. The main advantage of the redundancy in the ion position decoding by three coils (at 60° to each other) is the possibility of resolving simultaneous ion hits – crucial in ion–ion coincidence analysis, for example (Jagutzki *et al.*, 2002 ▸). The NIM-level output timing pulses are then fed to the HPTDC8 board in the control computer. The *X* and *Y* signals are routed via logical OR gates, although these are only needed for coincident operation. Also the MCP pulses are fed to the HPTDC8 board via an OR gate. The board records the arrival times of all these timing pulses relative to the trigger (‘Trig’) signal that, for pulsed-mode ions-only spectroscopy, is generated by the ‘Quantum Composers’ TTL pulse generator and then converted to a NIM level. The trigger is routed through a delay generator but, again, this becomes necessary only in coincident detection. The trigger signal is split to provide a simultaneous trigger pulse for the high-voltage extraction pulse generator (PVM-4210 model from Berkeley Nucleonics Co.). The various timing pulses collected by the HPTDC8 board are processed by the proprietary customized ‘*CoboldPC*’ software from Roentdek GmbH, which eventually stores the results – either processed ion TOF and position, or raw timing hits – of a measurement in various file formats (.bin, .lmf), that can be later processed by other software. For MUSTACHE, an analysis software package was developed and is available in Igor Pro 9 environment (Wavemetrics Co).

An electron–ion coincidence measurement is also handled by the *CoboldPC* software and the HPTDC8 acquisition board. For this, the following changes need to be implemented in the basic ions-only acquisition: (i) triggering the acquisition by electron signals while reducing the delay in extraction pulse generation, (ii) recording the electron position signals, and (iii) providing and identifying the ‘false coincidence’ triggers. These are implemented as follows:

(i) The complementary (analog) output from the electron MCP signal’s preamplifier is routed to the ‘Fast TD2000’ discriminator, whose output (with the discriminator level suitably adjusted) could, in principle, trigger the ‘Quantum Composers’ TTL pulse generator, which in turns triggers the ion extraction and acquisition. However, the TTL generator adds a significant 150 ns delay between the trigger and the output. To mitigate this, a delay generator unit, LeCroy 222, is used to raise the trigger to the HV pulse generator with a much smaller delay after the short electron MCP pulse arrives in its input. Simultaneously with the delay generator, also the TTL pulse generator is triggered by the electron signal discriminator. Here, the function of the delay generator is to keep the trigger level of the HV pulse generator HIGH after the electron discriminator’s signal, until the 150 ns delayed output of the TTL generator appears in the second ‘OR’ input of the delay generator. The HV trigger level is then maintained HIGH, as determined by the TTL generator’s pulse duration (typically 20 µs). The second output of the delay generator is used to trigger the acquisition in the HPTDC8 board.

The rising and falling edges of the HV pulse cause substantial electronic noise that affects not only the ion but also the electron detection. Therefore, spurious signals in the electron MCP circuit are likely to occur after these edges. These would trigger another ion extraction, which in turn causes spurious signals *etc*. – an infinite loop would be created. Such loops are prevented by the third output of the TTL pulse generator (channel ‘C’) that is used as VETO for the electron MCP discriminator and is set, typically, for 10 µs longer than the duration of the extraction pulse (determined by channel ‘A’).

(ii) The electron position signals can be combined with the ion position signals, taking advantage of the fact that they arrive in a different time window. Namely, the fastest detectable ions arrive after the noise from the rising edge of the extraction pulse has subsided, typically about 1 µs after the trigger. Until that time, the input of the HPTDC8 board is vetoed by software and configuration settings. However, even after eliminating the delay from the TTL generator, there is still about 200 ns delay between the trigger of the HPTDC8 board until the noise appears (due to the delay in the HV pulser unit). Therefore, there is a noise-free 200 ns time window in the ion signal channels, sufficient for electron position signals that arrive around the trigger time. They are taken from the auxiliary TTL output of the THR08 TDC unit and are added to the ion *X*_1,2_ and *Y*_1,2_ signal channels by the OR gates. They are then separated again and processed by a customized *CoboldPC* software and, in the final analysis, the time differences *T*(*X*_2_) − *T*(*X*_1_) and *T*(*Y*_2_) − *T*(*Y*_1_) are converted into the electron energy and the nondispersive position coordinate, respectively. Since the control software of the Scienta Omicron spectrometer is bypassed completely, various detector calibration operations are also performed during that analysis stage.

(iii) In the pulsed-mode operation, ‘false’ electron–ion and ion coincidences, which occur when the two seemingly coincident particles actually originate from different ionization events, can pose a significant problem. False coincidences can be reduced with very low ionization rates, although this is often unfeasible in practice. An effective remedy is to record a concurrent dataset containing *only* the false coincidences; it can be obtained by a ‘false’ electron trigger generated by a separate pulse generator (NIDAQ USB-6211) at a preset constant frequency (typically similar to the electron trigger rate). With false triggers, only ions that are present from random ionization from earlier events within a certain time window are collected; these would also provide the false coincidence background to the true electron-triggered events. The ‘false’-triggered dataset that runs concurrently with the electron-triggered acquisition can be subtracted from various types of histograms in later analysis. In order to identify a false-triggered event, in principle the missing electron position information in the *X*_1,2_ and *Y*_1,2_ channels suffices. However, in MUSTACHE, additional certainty is provided by the timing of a dedicated ‘trigtype’ signal (TTL generator’s output ‘B’) that is OR’d into the ion MCP channel. This signal takes advantage of the presence of the delay generator in the trigger circuit; in case of false triggers it arrives at the HPTD8 input near-simultaneously with the trigger, but for electron triggers it is delayed by about 150 ns, the time it takes for the triggered TTL pulse generator to produce output.

## 4. Performance and test results

Here we illustrate the MUSTACHE system’s performance with examples from simple systems such as argon, N_2_ and CS_2_, chosen to highlight the key aspects: (i) electron-energy-resolved electron–ion coincidences with high-energy Auger electrons from deep core ionization, (ii) ion-momentum-resolved coincidences with high-kinetic energy photoelectrons, and (iii) multi-ion coincidences following the decay of deep core ionization in molecules and Coulomb explosion.

### 4.1. Coincindences with energy-resolved Auger electrons

**Figure 4:**
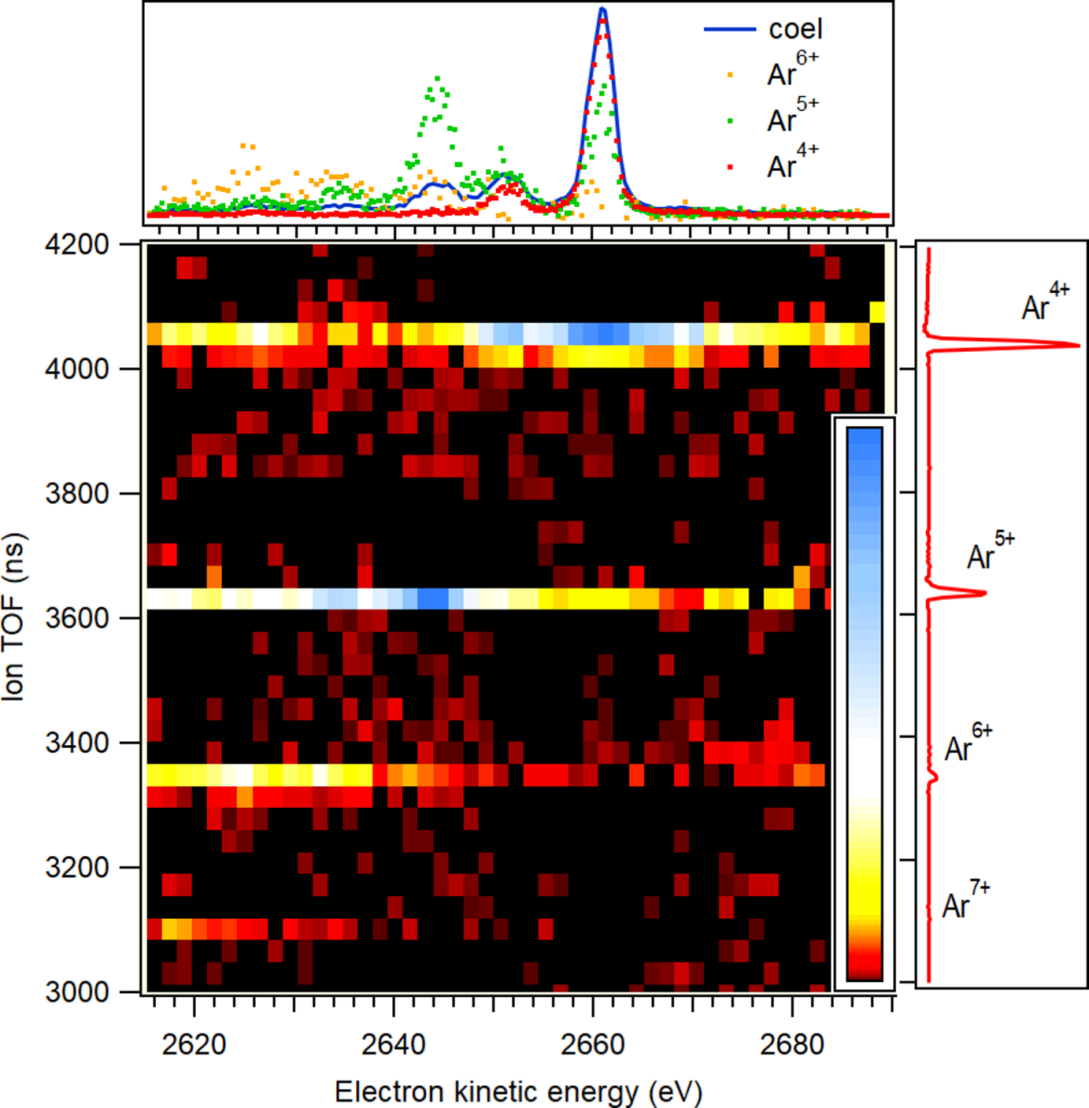
Main panel: PEPICO map of Ar^*n*+^ ions in coincidence with the *KLL* Auger electrons, following 1*s* ionization by 4.9 keV photons. The top panel shows the coincident electron spectrum (solid line, ‘coel’) and coincident ion yields for the Ar^4+^–Ar^6+^ ions (dots, ‘Ar^*n*+^’). The curves are scaled differently to improve visibility. The right-hand panel shows the coincident ion spectrum, from which the false coincidences were subtracted.

The Ar *KLL* PEPICO map, presented in Fig. 4 ▸, reveals different correlations of Ar^*n*+^ charge distributions with electron kinetic energies. The top panel of Fig. 4 ▸ shows the coincident Auger electron spectrum in the high kinetic energy range, retaining sufficient resolution to resolve spectral structures, and with fitted peak width starting from 250 meV. The dotted curves correspond to filtering by a specific ion and can be interpreted either as coincident ion yields or as ion-filtered electron spectra.

The strongest peak, at 2660.5 eV, and the smaller peak, at 2650.5 eV, correspond to direct transitions in both ionization and decay, *i.e.* (1*s*)^−1^ → (2*p*)^−2^ transitions, also known as diagram ^1^*D*_2_ and ^1^*S*_0_ lines, respectively (Püttner *et al.*, 2020 ▸). These transitions predominantly result in Ar^4+^, as the (2*p*)^−2^ double-core-hole states decay primarily via two sequential *LMM* Auger processes.

On the other hand, the peak at 2645 eV and a smaller peak at ∼2634 eV strongly correlate with Ar^5+^, which confirms their assignment as transitions from the Ar 1*s* satellites formed by shake-up and shake-off processes in direct ionization: (1*s*)^−1^(3*p*)^−1^(4*p*)^1^ → (2*p*)^−2^(3*p*)^−1^(5*p*)^1^ and (1*s*)^−1^(3*p*)^−1^(5*p*)^1^ → (2*p*)^−2^(3*p*)^−1^(6*p*)^1^ as well as (1*s*)^−1^(3*p*)^−1^ → (2*p*)^−2^(3*p*)^−1^, respectively (Püttner *et al.*, 2020 ▸). The PEPICO map shows that Auger decay paths from ionization satellites lead mainly to Ar^5+^ ion, indicating that the electron, shaken up during the ionization process to a higher valence orbital, has a very high probability to be ionized either by shake-off or valence Auger decay in the later stages of the Auger decay cascade.

### 4.2. Coincidences with high-energy photoelectrons and ion momentum resolution

#### 4.2.1. N_2_ molecule, high- and low-momentum ions

**Figure 5:**
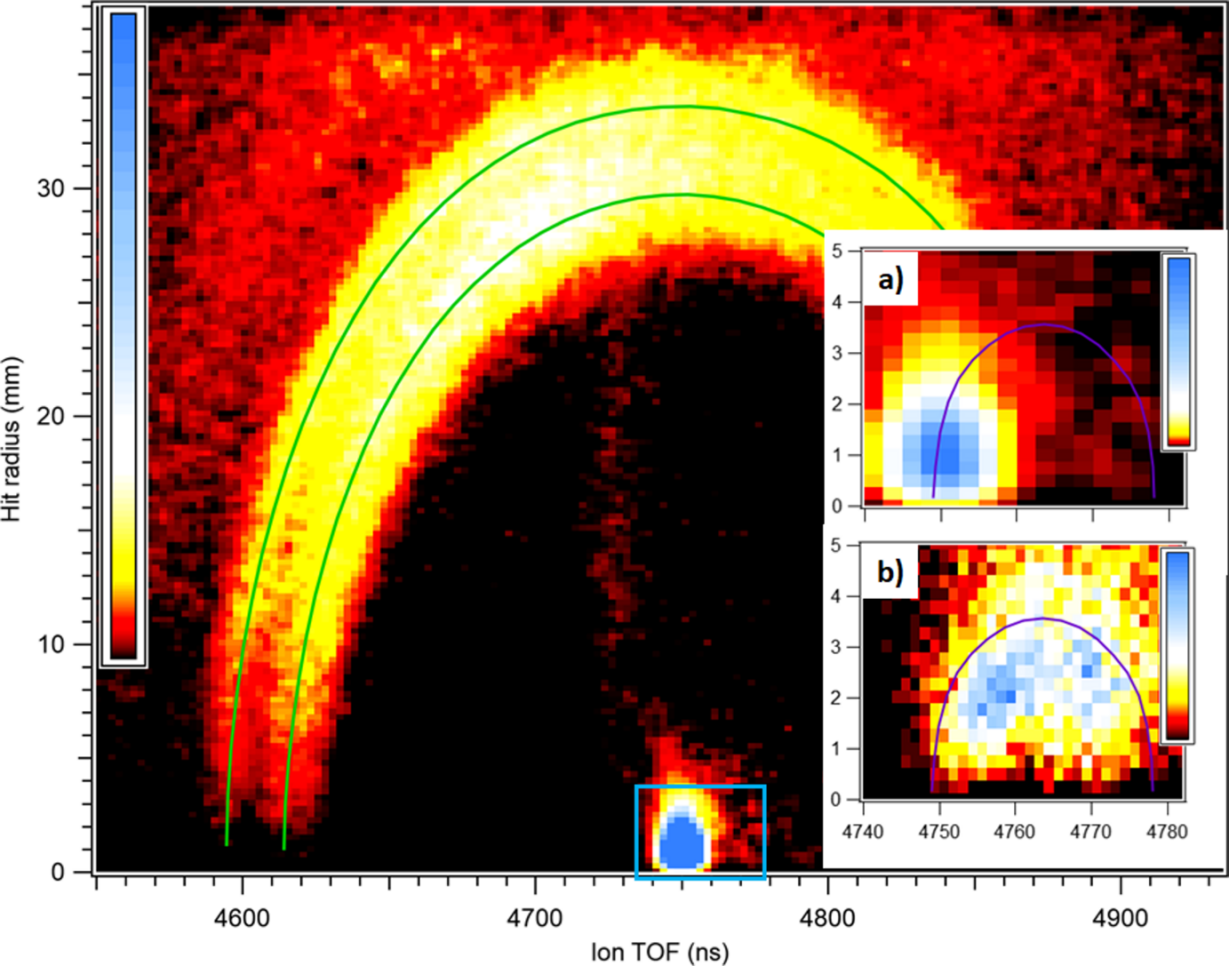
Main panel: 2D false-color map of ion hit radius *R* versus TOF for the N^+^ and 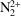 ions, from a PEPICO measurement with N 1*s* photoelectrons of N_2_ at *hv* = 4.9 keV. A false coincidence 2D map has been subtracted. The blue rectangle marks the region of the 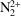 ions, shown in detail in inset (*a*). Inset (*b*) shows this region for the false-triggered events. Solid lines represent simulated *R*(TOF) curves for N^+^ with 3.75 and 4.95 eV kinetic energy (green) and for 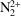 with 176 meV of recoil energy (magenta).

Fig. 5 ▸ illustrates the system’s capabilities for momentum-resolved coincidence measurements with photoelectrons of high kinetic energy, in both low and high momentum range. We have chosen N_2_ as a testbed system, that was also used in a corresponding commissioning work with a PEPIPICO endstation at the soft X-ray beamline FinEstBEAMS at the MAX IV synchrotron (Kooser *et al.*, 2020 ▸; Pärna *et al.*, 2017 ▸). In the present case, the N 1*s* electrons were ionized by 4.9 keV photons, under the high-kinetic-energy photoemission regime. The electron energy resolution in the coincidence data is used here firstly to eliminate contributions from valence ionization and secondly to filter out satellite transitions at the lower kinetic energy than the main N 1*s* photoline. This does not require the highest possible electron resolution, which was set to ≲1 eV at 200 eV pass energy of the analyzer.

**Figure 6:**
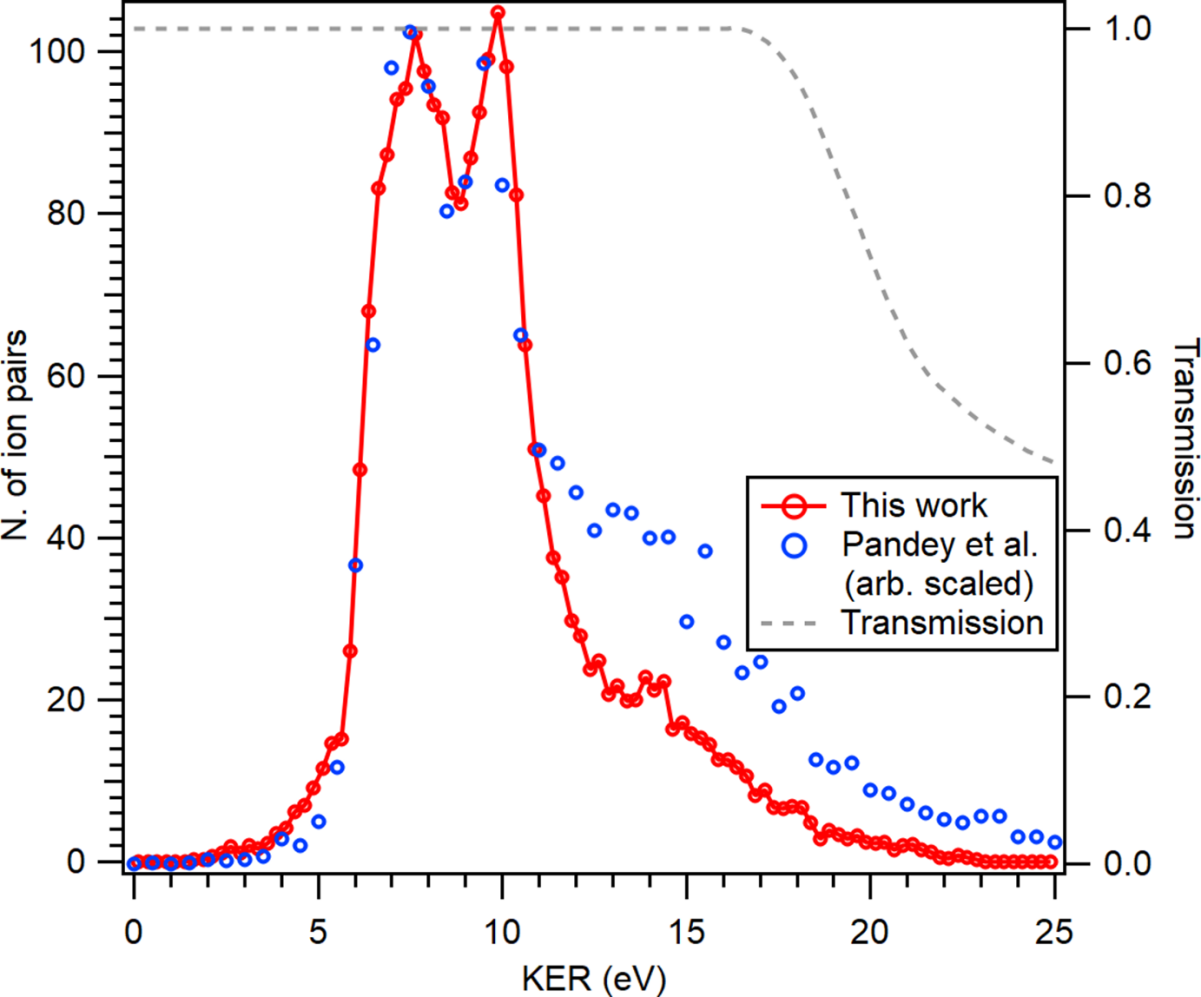
Kinetic energy release (KER) determined from a PEPIPICO measurement at *h*ν = 4.9 keV, with energy-selected N 1*s* photoline in the 4486–4495 eV kinetic energy window. Open circles are a digitized result from Pandey *et al.* (2016 ▸), measured using 5 keV electron impact. The dotted gray line shows the ion transmission as a fraction of the full solid angle.

The main panel of Fig. 5 ▸ shows a 2D map of N^+^ coincident ions’ (resulting from the dicationic dissociation 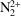 → N^+^ + N^+^) hit radius on the detector *versus* their TOF. From the former, the radial components of the ion momenta can be derived, and, from the latter, the axial components (with reference to the spectrometer’s axis). However, in the plot we have chosen to show the raw, measured data. The map is constructed from photoelectron-triggered events, but a false coincidence map from the events by ‘false’ triggers (Section 3) is subtracted, removing the contribution from ions accidentally present in the interaction region during the detected photoionization event. The graph shows a double arc that arises from the N^+^ ions that, due to dicationic dissociation, have achieved significant kinetic energy. After applying the necessary conversion factors, obtained with the aid of ion ray-tracing simulations (such as in Fig. 2 ▸), we made a selection of (N^+^, N^+^) ion pairs with momentum filtering (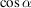 < −0.9, where α is the angle between the two momenta), also restricting the coincidences only to the main N 1*s* photopeak in the coincident electron spectrum. The obtained kinetic energy release (KER) is shown in Fig. 6 ▸, and is compared with literature results (Pandey *et al.*, 2016 ▸). We can see that the two double arcs of Fig. 5 ▸ correspond to the maxima of the KER at 7.5 and 9.9 eV. There is an excellent agreement with Pandey *et al.* up to about 10 eV; beyond that our experiment shows a lower fraction of high-KER events. The difference can be partly due to the decreased transmission for high-kinetic-energy ions that are not collected from the full 4π solid angle of the initial velocity distribution anymore. This effect was quantified by ion ray-tracing simulations, with the estimated transmission as the dotted gray line in Fig. 6 ▸, showing a decrease above about 15 eV KER. Note, however, that if the ion image is not perfectly centered, the transmission starts to be decreased at a lower KER. Our result can also be compared with a high-resolution measurement (Kircher *et al.*, 2019 ▸), where a marked change in the high-KER events when using different ionizing photon energy (419 eV *versus * 40 keV) was observed. Thus, differences with Pandey *et al.* can also be attributed to the different ionization conditions.

Near the center of the arc is a well defined bright spot of the slow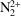 parent ions. Uniquely to this measurement in comparison with test measurements of soft X-ray setups (Kooser *et al.*, 2020 ▸), here the ion receives a significant *recoil momentum* in the opposite direction of the departing photoelectron due to its high kinetic energy. The 4.4 keV photoelectrons have a momentum of 18.2 a.u. and thus give equal translational recoil momentum to the 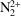 dication. This equals to 176 meV of translational energy of 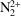, about 25 times less than the energy of the N^+^ fragments from the Coulomb explosion. According to the geometry of MUSTACHE (Fig. 1 ▸), the opposite direction of the *detected* coincident photoelectron is towards the ion detector, and therefore the added recoil momentum *shortens* the ion flight time. Indeed, 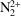 is shifted from its calibrated position – the center of the half-circle in panels (*a*) and (*b*) of Fig. 5 ▸ – by about 14 ns (note that the arc of N^+^ ions is equally shifted). A comparison is provided by the analogous image in panel (*b*) that is created from false-triggered events: that is, an electron was not actually detected and essentially a non-coincident single-ion measurement was performed. In this case, the direction of the departed photoelectron is unknown, and therefore the recoil momentum of the detected 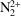 ion can have any direction, with the axial component shortening or lengthening the flight time and with the radial component causing deviation from the spectrometer’s axis. Inset (*b*) shows a small-sized arc corresponding to this recoil momentum, which is compared with ray-tracing simulations that are represented by a half-circle. The simulations were performed for 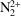 ions having the initial randomly oriented momentum equal to the recoil momentum.

We see that the spectrometer can simultaneously achieve good ion momentum resolution across both low and high momentum ranges, in coincidence with electrons of high kinetic energy. For Fig. 5 ▸, the ion extraction, lens and acceleration voltages were ±230 V, −200 V and −875 V, respectively. The momentum resolution can be improved with lower operation voltages, but then the solid angle of collection of the high-momentum ions will become less than 4π. The size of the interaction region can be an important limiting factor of momentum resolution in all three dimensions. It is expected to be the largest in the radial direction, along the photon beam, where its size is determined by the expansion of the gas jet, from a 500 µm inner-diameter needle in this case, about 5 mm from the photon beam. The lens voltage in this measurement results in a radial source magnification coefficient of only 0.16 (from ray-tracing simulations); thereby a 5 mm source is reduced to a less than 1 mm spot on the detector. The 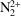 spot size in Fig. 5 ▸(*a*) is further broadened by the spatial resolution of the detector itself and by the thermal velocity of the molecules at room temperature.

In principle, with further improved momentum resolution (*e.g.* by sample cooling and more collimated sample jet), the recoil-giving photoelectron’s angular distribution could be derived from a non-coincident ion momentum measurement such as shown in panel (*b*) of Fig. 5 ▸, although we note that MUSTACHE is not optimized for such types of experiment. In the present case, the distribution of the 1*s* photoelectrons peaks in the horizontal direction along the polarization vector (corresponding to the maximum deviations of ion TOF), and drops to zero in the vertical direction (at the largest ion hit radius). This is in general agreement with panel (*b*) already at present resolution, where we see decreased intensity near the maximum radius that corresponds to recoil momentum and therefore also the photoemission perpendicular to the polarization vector. In contrast, in the case of isotropic emission, one expects maximum intensity in the ion distribution in the perpendicular direction (according to the geometrical 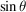 factor).

#### 4.2.2. Argon ions with photoelectron recoil momentum

**Figure 7:**
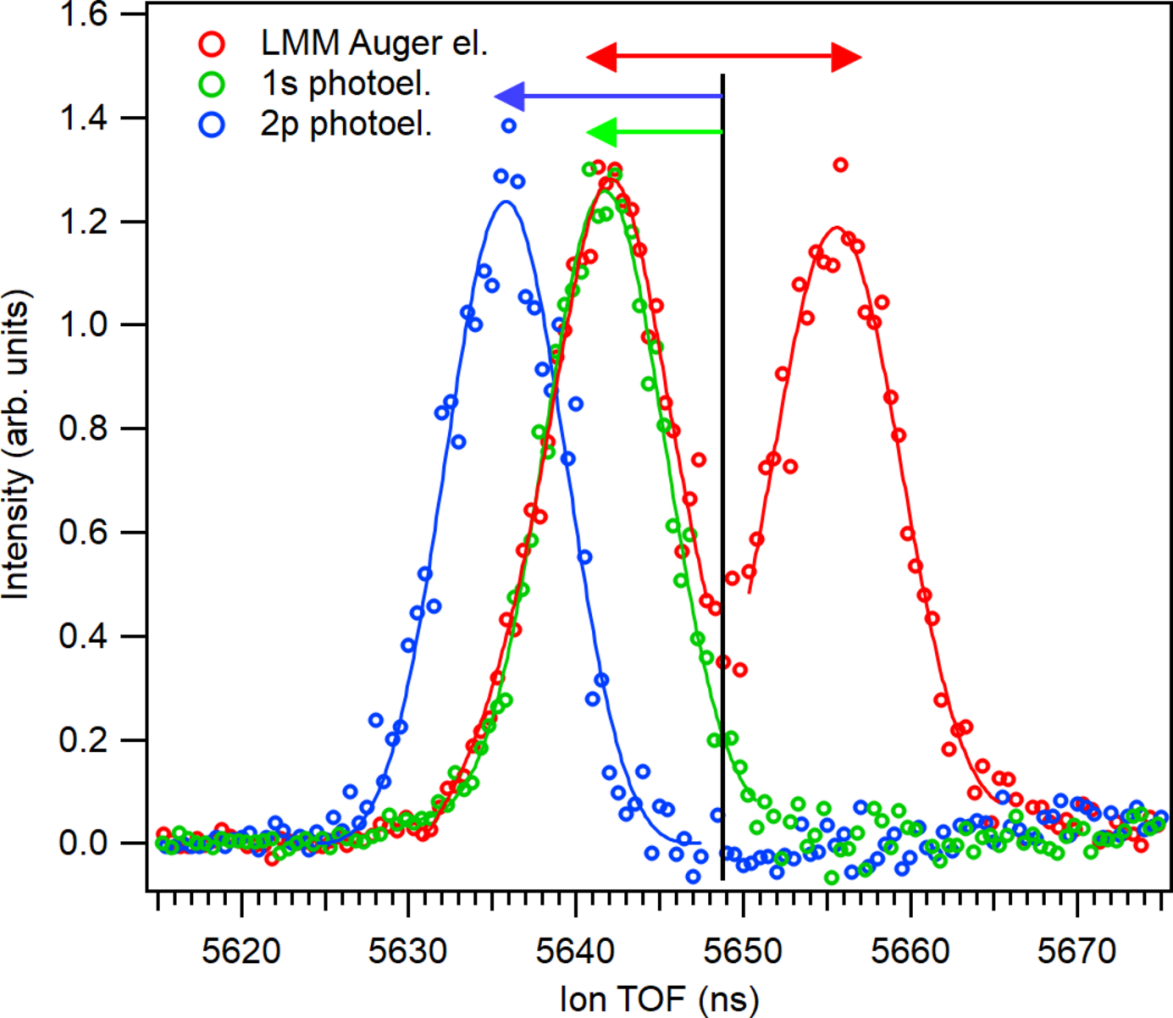
Ion TOF spectra of Ar^2+^ ions, measured at 4.9 keV photon energy in coincidence with Ar 1*s* photoelectrons (green markers); Ar 2*p* photoelectrons (blue) and Ar *LMM* Auger electrons (red). False-coincidence spectra were subtracted from the data points. Solid lines are Voigt-curve fits to the data points. The black vertical line marks the nominal peak position without recoil shift and the arrows of corresponding color indicate the predicted recoil shift.

In this example, PEPICO measurements were performed at a photon energy of 4.9 keV, with various emitted electrons having kinetic energy up to 4.65 keV, approaching the upper limit of MUSTACHE’s energy range. Similar to the case of 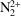 ions, the Ar^2+^ ion TOF spectra of Fig. 7 ▸, recorded in coincidence with electrons from different processes, exhibit the effects of photoelectron recoil. The component of the recoil vector along the main axis induces shifts in the ion TOF, as observed in the figure. To explore how different types of coincidences manifest in the recoil shift, we first recorded an ion spectrum in coincidence with the *LMM* Auger electrons. These electrons, emitted when a 2*p* hole is filled, have relatively low kinetic energy (∼200 eV), resulting in a small recoil momentum. However, the 2*p* hole itself is created either by direct photoionization of the 2*p* orbital or through cascade or fluorescence processes that follow 1*s* photoionization. In the latter case, a higher-energy electron is emitted, which imparts a larger recoil momentum. The argon dications Ar^2+^ in Fig. 7 ▸ cannot originate from a *KLL*-cascade Auger process, which would primarily lead to Ar^4+^, leaving only two pathways: direct 2*p* ionization or 1*s* ionization followed by *K*_α_ decay (Guillemin *et al.*, 2018 ▸). This implies that these events are necessarily accompanied by an unobserved high-kinetic-energy photoelectron. The recoil energy of Ar^2+^ ions due to the 1*s* photoelectron, which has a kinetic energy of 1675 eV, is about 64 meV and the recoil momentum is 11.1 a.u., though its direction remains undetermined. The angular distribution of the 1*s* photoelectron, characterized by an anisotropy β ≃ 2, results in the majority of electrons being emitted along the main axis when using a horizontally polarized incident photon beam – either towards or away from the ion detector – while vertical emission probability drops to zero. This leads to a symmetric two-peak structure, as seen in Fig. 7 ▸, arising from the recoil momenta of the ions directed parallel (towards the detector) and anti-parallel (away from the detector) to their flight direction. The red arrow indicates the calculated splitting due to a recoil momentum of ±11.1 a.u., as determined from ΔTOF values obtained via ray-tracing simulations.

Next, the measurement was repeated in coincidence with the Ar 1*s* photoelectrons. The key difference, in this case, was that the recoil-inducing electron was detected, thereby determining the direction of the recoil momentum. As observed for N_2_, the coincident ions now experience recoil only *towards* the detector, leading to a reduced flight time. Consequently, a single shifted peak is observed in Fig. 7 ▸.

Finally, the coincident electron detection window was adjusted to capture the 2*p* photoelectrons, which have a kinetic energy of 4650 eV at a photon energy of 4.9 keV. The resulting recoil momentum is now larger, 18.5 a.u., with the shift still directed exclusively towards the shorter TOF but with an increased magnitude. Similar to the case of coincidences with the 1*s* photoelectron, a single recoil-shifted peak is observed. By comparing the shifts in Fig. 7 ▸, we conclude that the recoil in the *LMM* Auger decay is indeed induced by the 1*s* photoelectron, confirming that 1*s* ionization followed by *K*_α_ fluorescence is the dominant pathway forming this spectrum. At the photon energy of 4.9 keV, the cross section of 1*s* ionization is significantly higher than that of 2*p*, further supporting this interpretation.

### 4.3. Momentum-resolved multi-ion coincidences following deep core ionization and Coulomb explosion

**Figure 8:**
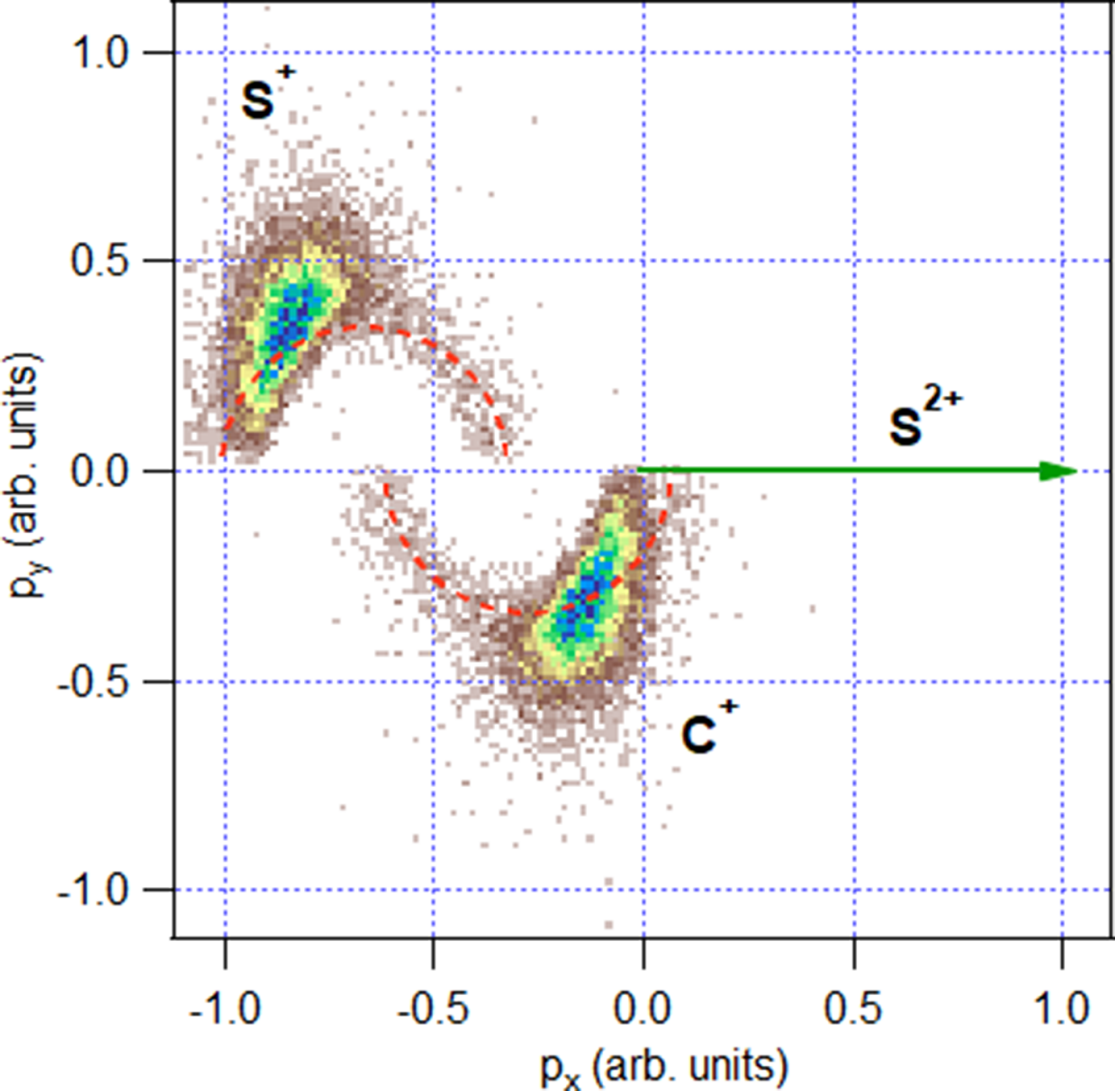
Newton diagram for events of the three-body dissociation of CS^4+^ into a (S^2+^, C^+^, S^+^) ion triplet, in coincidence with the S 1*s* photoelectron (main line and satellites), following ionization of neutral gas-phase CS_2_ molecules. The momentum of the S^2+^ ions is normalized to a unit vector along the *x*-axis. The ion triplets from all electron-triggered events were filtered for the momentum sum less than 30.1 a.u., efficiently eliminating false coincidences. Red dashed lines serve as visual guides for the minor sequential dissociation channel.

The ion momentum imaging capabilities of MUSTACHE underwent testing through an examination of the extensively studied case involving the three-body dissociation of CS_2_ subsequent to S 1*s* ionization (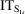 ≃ 2.5 keV). Fig. 8 ▸ displays a Newton diagram illustrating the momentum distribution subsequent to data filtering for the predominant dissociation channel of S^2+^/C^+^/S^+^. Newton diagrams are graphical representations used to analyze the momenta of several fragments produced in the dissociation event. These diagrams showcase the momentum vectors of the particles involved in a process. Each vector’s length signifies the ion’s momentum magnitude, while the angle indicates its direction relative to a chosen reference frame, *i.e.* momentum of the S^2+^ ion. The prominent spots in the diagram are associated with concerted fragmentation, indicating simultaneous bond breakage at the molecular geometry close to the equilibrium ground-state geometry of CS_2_. The faint semi-circular patterns, marked by red dotted lines as a guide in Fig. 8 ▸, correspond to a minor sequential dissociation channel (Neumann *et al.*, 2010 ▸; Wales *et al.*, 2014 ▸; Rajput *et al.*, 2018 ▸; McManus *et al.*, 2022 ▸). In this channel, the fragmentation occurs in two steps: first, the formation of the S^2+^ atomic fragment, and then the dissociation of the remaining CS^2+^ into C^+^ and S^+^ ions, when the S^2+^ ion has moved sufficiently far to no longer exert any influence. The momentum correlation diagram obtained from the data recorded using MUSTACHE (Fig. 8 ▸) mirrors those obtained previously using COLTRIMS coincidence setups (Guillemin *et al.*, 2015 ▸; Wang *et al.*, 2018 ▸; Guillemin *et al.*, 2022 ▸). The semi-circular patterns, indicative of the minor sequential, or stepwise, dissociation, are faithfully replicated in our dataset, achieved within a relatively brief data acquisition period of approximately four hours. Sequential fragmentation, competing with charge redistribution, is described as localized, contrasting with the concerted fragmentation where bond breakage happens simultaneously after rapid charge redistribution within the molecule. Understanding the interplay between charge redistribution and fragmentation is pivotal in studies of radiation damage, where detailed insights into energy distribution are important.

## 5. Conclusion

In conclusion, the MUSTACHE setup demonstrates its effectiveness in high-resolution electron–multi-ion coincidence measurements, offering a versatile approach for studying complex photoinduced processes in the gas phase. The system’s design, including its ability to handle both soft and hard X-ray photon energies, ensures broad applicability to various molecular systems, while the initial test results highlight its potential for exploring fundamental aspects of deep-core ionization, Auger decay cascades, recoil, and Coulomb explosion phenomena. This setup paves the way for further advancements in hard X-ray-based spectroscopic techniques.

Detection of fast electrons while retaining good energy resolution, combined with multi-ion detection, is particularly valuable for studying charge redistribution processes during Auger cascades. The energy range accessible with MUSTACHE approaches the hard X-ray regime, making it highly effective for investigating molecular responses to ionization sources, including those employed in medical applications. This is particularly relevant for high-*Z* elements, such as iodine-containing compounds, where the predominant fluorescence decay [88% (Krause, 1979 ▸)] of the *K*-core-hole state creates an *L*-shell vacancy, triggering a cascade of Auger decays. These secondary processes, which fall within the energy range of the setup, provide an effective model for studying high-energy X-ray interactions at the molecular level. By simulating radiation exposure conditions found in biological environments, MUSTACHE can provide insights into the early stages of radiation-induced damage, with potential applications in improving radiotherapy techniques and the development of effective radiosensitizers.

## Data Availability

The data supporting the results reported in the article can be accessed upon reasonable request from the corresponding authors.
